# Stabilization of an Aqueous Bio-Based Wax Nano-Emulsion through Encapsulation

**DOI:** 10.3390/nano12234329

**Published:** 2022-12-06

**Authors:** Pieter Samyn, Vibhore K. Rastogi

**Affiliations:** 1 Chair of Bio-Based Materials Engineering, University of Freiburg, Werthmannstrasse 6, D-95070 Freiburg, Germany; 2 SIRRIS—Department Circular Economy and Renewable Materials, Wetenschapspark 3, B-3590 Diepenbeek, Belgium; 3 Department of Paper Technology, Indian Institute of Technology Roorkee, Roorkee 247667, Uttarakhand, India

**Keywords:** biowax, emulsion, encapsulation, nanoparticles

## Abstract

The emulsification of biowaxes in an aqueous environment is important to broaden their application range and make them suitable for incorporation in water-based systems. The study here presented proposes a method for emulsification of carnauba wax by an in-situ imidization reaction of ammonolysed styrene (maleic anhydride), resulting in the encapsulation of the wax into stabilized organic nanoparticles. A parameter study is presented on the influences of wax concentrations (30 to 80 wt.-%) and variation in reaction conditions (degree of imidization) on the stability and morphology of the nanoparticles. Similar studies are done for encapsulation and emulsification of paraffin wax as a reference material. An analytical analysis with Raman spectroscopy and infrared spectroscopy indicated different reactivity of the waxes towards encapsulation, with the bio-based carnauba wax showing better compatibility with the formation of imidized styrene (maleic anhydride) nanoparticles. The latter can be ascribed to the higher functionality of the carnauba wax inducing more interactions with the organic nanoparticle phase compared to paraffin wax. In parallel, the thermal and mechanical stability of nanoparticles with encapsulated carnauba wax is higher than paraffin wax, as studied by differential scanning calorimetry, thermogravimetric analysis and dynamic mechanical analysis. In conclusion, a stable aqueous emulsion with a maximum of 70 wt.-% encapsulated carnauba wax was obtained, being distributed as a droplet phase in 200 nm organic nanoparticles.

## 1. Introduction

Bio-based waxes are increasingly applied in industry as a renewable replacement for traditional fossil-based waxes, with favorable properties in mechanical protection, scratch resistance, hardness, thermal stability and hydrophobicity [1]. The biowax composition contains a more complex mixture of molecular chains than synthetic waxes, including substituted long-chain aliphatic hydrocarbons with alkanes, alkyl esters, fatty acids, primary and secondary alcohols, diols, ketones and aldehydes [2]. The variation in chemical functionalities and molecular weight finally determines the ultimate properties, while specific physical properties cannot uniquely be correlated to the melting point [3]. Fossil-based waxes (e.g., paraffin wax) are usually produced as a by-product of oil refining and distillation, and the properties for industrial use are strongly affected by a residual oil content [4]. Paraffin wax has alkane molecules with between 20 and 40 carbon atoms and a molecular weight that is usually less than half that of most polyethylene waxes. Therefore, paraffin wax usually has a much lower melt point and is softer than polyethylene waxes [5]. The synthetic Fischer-Tropsch wax is alternatively obtained from the coal chemical industry and consists of saturated linear polymer chains of fully saturated alkanes with lower molecular weight than polyethylene wax [6], providing a more crystalline structure with consequent oxidation resistance, high melting point, narrow melting point range, low oil content, low needle penetration, low mobility, low melt viscosity, high hardness, wear resistance and high stability [7]. In contrast, natural waxes originate from animals (e.g., beeswax, shellac wax) [8], or plants (e.g., carnauba wax, also known as palm wax, candelilla wax, soybean wax, castor wax, rice bran wax, sunflower wax) [9], where it forms a waterproof layer and reflects solar UV radiation on the leaves to protect against drought [10]. The plant wax is present as a component in the thin amorphous epicuticular layer [11], where it protects and provides self-cleaning properties [12]. In industrial use, the different properties of biowaxes mean they are potentially included in a range of products, such as the following: protective coatings, paints and inks (e.g., anti-blocking properties, anti-graffiti, gloss improvement, slip modification, hydrophobicity) [13]; protective edible coatings (e.g., barrier properties, anti-microbial, shelf-life) [14,15]; food packaging (e.g., barrier properties, light protection) [16]; composites (e.g., interface reinforcement) [17]; construction (e.g., phase change materials) [18]; energy conversion and management (e.g., thermal energy storage) [19]; metal protection (e.g., lubricity, corrosion protection) [20]; paper coating (e.g., barrier properties, heat sealability) [21]; textile finishes (e.g., gloss improvement, softness, water repellence) [22]; cosmetics (e.g., skin care) [23]; food (e.g., microencapsulation or food coating) [24]; wood products (e.g., hydrophobization, slip, abrasion resistance) [25]; or in the machining industry (e.g., bio-lubricant) [26]. For example, the hydrophobicity of paper and wood coatings with carnauba wax improved with water contact angles of over 140° [27]. The efficiency in barrier dispersion coatings for paper containing different natural and paraffin was comprehensively studied, confirming that biowax coating agents could significantly improve the hydrophobicity (by almost double) and reduce air permeability (by almost thrice) compared with paraffin dispersions, possibly because of lower viscosity [28]. For coating applications, the supply of bio-based wax additives in a stable aqueous emulsion (oil-in-water O/W emulsion) is preferred for environmental reasons. and compatibility with running coating formulations, processing and machinery.

The emulsification of wax in a continuous water phase involves the dispersion of wax as an immiscible phase while not forming a thermodynamic equilibrium. The phase interface can artificially be created under mechanical energy (e.g., shear) and needs to be stabilized by surfactants to prevent re-agglomeration and coalescence over long storage periods. O/W emulsions with soybean wax were stabilized with different emulsifiers. such as ethylene glycol and propylene glycol mono- and di-esters of stearic acid, respectively, resulting in particle sizes of 20 to 100 µm, depending on the type of commercial stabilizer [29]. Petroleum resin resulted in a good emulsification of paraffin wax within 30 min at 90 to 95 °C and controlled stirring speed, providing wax emulsions with monodisperse particle size [30]. The melt dispersion technique was used for production of carnauba wax microparticles, where particle size and morphology were controlled by wax concentration, stirring speed, stirring time, and surfactants [31]. The long-term stability of dispersions after melt emulsification of candelilla wax in the presence of commercial emulsifiers was demonstrated in [32], where the hydrophilic–lipophilic balance of the surfactants was a critical parameter for stabilization. Viscosity and chemical compatibility have particular effects on particle sizes of emulsified wax, which, however, still remain in the micrometer size range (100 to 150 µm). However, the amount of wax that can be emulsified in water remains limited, depending on the preparation conditions and surfactant. For example, carnauba wax emulsion exhibited the best emulsifying effect and the smallest particle size when the mass fraction of carnauba wax was 10%, the emulsifier mass fraction was 1.5% and the rotation speed was 500 r/min [33]. Alternatively, submicron paraffin wax emulsions with droplets of 700 nm were prepared by the emulsion inversion point method in [34], as mixing all the components together, or the addition of oil to the aqueous surfactant dispersion, did not produce stable emulsions. Emulsions with micrometer particles of beeswax were stabilized in a chitosan matrix prepared from acetic acid solution, using glycerol as a plasticizer and heating above the wax melting temperature [35]; however, phase separation occurred after several days. A two-step homogenization procedure was further optimized to make paraffin and beeswax dispersions with stearates, stearic acid or glucoside emulsifiers at alkaline pH, where wax nanoparticles of 350 to 440 nm were electrostatically stabilized, depending on the type of hydrophobic wax [28]. In general, preparation of concentrated nano-emulsions requires high energy input under extreme mechanical shear [36]. Alternatively, they can be obtained without external energy while using the intrinsic chemical energy in the system: the low-energy methods include a phase-inversion temperature [37], or emulsion inversion point [38].

The encapsulation of biowax allows the production of capsules with a polymer shell at the outer side that provides additional mechanical strength and protection, against, for example, thermal degradation, or allows delivery of the wax under desired conditions. The polymer layer for micro-encapsulation can be formed through in-situ polymerization [39], interface polymerization [40], or complex coacervation [41]. As such, the synthesis of microscale capsules of paraffin wax was demonstrated through the absorption and in situ polymerization of a urea–formaldehyde prepolymer in the presence of a hydrolyzed styrene maleic anhydride copolymer as an emulsifier [42]. Although the copolymer provides efficient stabilization of the particle sizes, the residual emulsifier content has negative effects on the dimensional, thermal and mechanical stability of the polymer shell and can, possibly, cause fracture. The nanoscale encapsulation of wax was demonstrated by in-situ emulsion polymerization of a polystyrene–acrylate resin, where the homopolymer of styrene (St) and copolymer of styrene-butyl acrylate (St-BA) indicated small differences on the size of the composite particles, but a significant difference in thermal performance [43]. The nano-encapsulation of paraffin wax with melamine-formaldehyde (MF) as the shell, and synthesized by the in-situ polymerization method, is a favorable route for the production of regular spherical particles of 260 to 450 nm, where the encapsulation efficiency increased with the supplied amount of core material up to 75% [44]. After encapsulation of paraffin wax as a core within a polystyrene shell through mini-emulsion polymerization, however, it was observed that the thermal stability of the nano-capsules needed to be further optimized by the addition of crosslinking agents, influencing the crosslinking density of the polystyrene shell [45].

In this study, the emulsification of wax as nanoparticles in an aqueous environment was performed through the encapsulation of wax by a styrene–maleic anhydride copolymer. The latter forms a polymer shell around the wax droplets through self-organization at the interface and is further imidized to stabilize the copolymer as an organic phase containing the wax phase. The stabilization of the nanoparticles by in-situ imidization is critical to enhance the thermal stability and mechanical properties, but needs to be balanced with the stability of the emulsion. As an advantage, compared to the techniques mentioned above, the method presented does not require strong mechanical energy input, no additional surfactants are used that may have a negative effect on further mechanical properties, wax emulsions with smaller droplet sizes are obtained (nano-emulsions, in contrast to previously presented microscale droplets), having higher thermal stability (imidized organic phase) and no phase separation over a long storage time. A comparative study is made using paraffin and carnauba wax as two representative wax types for industrial applications, illustrating the benefits of carnauba wax as being more reactive and providing better compatibility with the imidization reaction.

## 2. Materials and Methods

### 2.1. Materials

**Figure 1:**
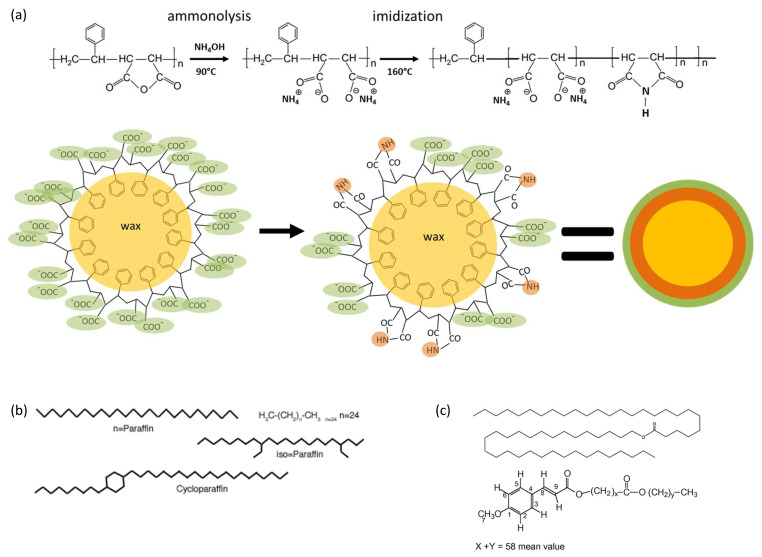
Schematic representation for the synthesis of wax nano-emulsions through encapsulation of carnauba wax or paraffin wax by an imidization reaction of styrene–maleic anhydride, (**a**) chemical synthesis reaction with first step of ammonolysis and second step of imidization, resulting in formation of stabilized wax nanoparticles, (**b**) chemical structure of paraffin wax, (**c**) chemical structure of carnauba wax.

Poly(styrene–maleic anhydride) copolymer, or SMA, with 26 mol-% maleic anhydride, 74 mol-% styrene and number-average molecular weight M_n_ = 80.000 g/mol was obtained from Polyscope (Geleen, The Netherlands) and used as ground pellets. Ammonium hydroxide was obtained from Belgocare (Niel, Belgium) and used as a 25% aqueous solution (0.9 g/mL). The wax emulsions were prepared with the incorporation of carnauba wax (CW, CAS 8015-86-9, Sigma Aldrich, Burlington, MA, USA), and a reference paraffin wax (PW, CAS 8002-74-2, Sigma Aldrich) with a melting point of T_m_ = 53 to 58 °C. An outline for the synthesis of stable wax emulsions in an aqueous environment, through encapsulation, is given in Figure 1, involving the ammonolysis and in-situ partial imidization of SMA into an organic shell. A representative chemical formula for waxes is inserted in Figure 1b,c. Paraffin wax consists of straight chain hydrocarbons with 80 to 90% normal paraffin content and the balance consists of branched paraffins (iso-paraffins) and cycloparaffins. Carnauba-wax has a more complex structure, with a mixture of wax esters, free fatty acids with long-chain alcohols, unsaturated hydrocarbons and, possibly, aromatic compounds, e.g., including aliphatic esters (40 wt.-%), diesters of 4-hydroxycinnamic acid (21 wt.-%), ω-hydroxycarboxylic acids (13 wt.-%), and fatty alcohols (12 wt.-%). Owing to the controlled ammonolysis reaction of the SMA copolymer, no additional surfactants were added.

The aqueous emulsions were prepared in an autoclave by varying the wax concentration (*w*/w) relatively to the SMA copolymer, i.e., wax/SMA = 0/100; 30/70; 50/50; 70/30 and 80/20. The reference material of wax/SMA = 0/100 corresponded to the synthesis of pure imidized SMA or styrene maleimide (SMI) nanoparticles as previously performed in [46]. The solid content (S.C.) of the emulsions was varied through changing the total weight of materials loaded relatively to the volume of water, i.e., S.C. = 50% or S.C. = 60%. The maximum volume of water in the autoclave was 1 L and an appropriate ratio of solid materials was added in order to achieve the target S.C. The imidization was carried out with a fixed ratio of ammonium hydroxide to maleic anhydride of 1/1.01, as optimized before in [46].

The wax emulsions were made by adding ground wax flakes together with SMA copolymer and ammonium hydroxide in the quantities mentioned before. The first part of the reaction was conducted while maintaining a temperature of 90 °C under 1 bar pressure for a time of about 1 h, allowing for the melting of the wax and ammonolysis of the SMA. The temperature was subsequently augmented to 160 °C under a pressure of 6 bar for 4 h, allowing for the partial imidization of the ammonolysed SMA into a polymer shell. The stirrer was continuously driven at a fixed speed of 400 rpm, while measuring the variations in viscosity in the reactor through the power. The strong drop in viscosity after a reaction time of 4 h indicated almost full conversion, after which the reactor was cooled down to room temperature. The emulsions were obtained as homogeneous white liquids with no signs of remaining solid fractions upon successful synthesis.

### 2.2. Characterization Methods

The quality of wax emulsions is determined by pH measurements (Knick 752 Cl, nr. 051489, Berlin, Germany), solid content (S.C.) determination through infrared drying and weighing (LP16/PM600, Mettler Toledo, Columbus, OH, USA), dynamic light scattering (DLS) (Zetasizer Nano ZS, Malvern Analytik Ltd., Cambridge, UK), Zetapotential (Zetasizer Nano ZS, Malvern Malvern Analytik Ltd., Cambridge, UK), and viscosity measurements (Brookfield, DV-II Pro, spindle n° 3, Bookfield Ametek, Harlow, Essex, UK). The S.C. was expressed as a weight fraction (%) of the dried material measured after drying of a 50 mL droplet. The DLS results were determined after 500 times dilution and reported as the z-average particle size (nm), while the Zetapotential (mV) was measured on the same diluted sample under the intrinsic pH of the emulsion. The emulsions were further diluted 100 times and air-dried overnight onto a carbon-grid for evaluation by transmission electron microscopy or TEM (Leo CEM 912, Zeiss, Jena, Germany), operating under an acceleration voltage of 100 kV.

The dried materials (48 h air drying) were characterized by differential scanning calorimetry or DSC (DSC 2910, TA Instruments, Waters, Antwerp, Belgium), thermogravimetric analysis or TGA (Pyris 1, Perkin Elmer, Rodgau, Germany), dynamic mechanical analysis or DMA (DMA 8000, Perkin Elmer), Fourier transform infrared spectroscopy or FTIR (Spectrum 65, Perkin Elmer, Rodgau, Germany), dispersive Raman spectroscopy (Raman Flex 400, Perkin Elmer, Rodgau, Germany). The DSC was performed on a sample of 5 mg under nitrogen atmosphere, heated in hermetically sealed pans between −50 to 250 °C with a heating rate of 10 °C/min, following two heating cycles with an intermediate cooling cycle at a cooling rate of 20 °C/min. The DMA tests were done by pressing 20 mg powder in between a stainless-steel envelope and mounting the bars as a single cantilever configuration. A dynamic bending mode was applied with one end of the pocket clamped to a fixed support and the other end clamped to the drive shaft undergoing a displacement of 0.05 mm with a frequency 1 Hz. The DMA tests were done by heating at 5 °C/min over a temperature range of 25 to 250 °C, while recording the modulus (E) and loss factor (tan δ). The TGA was done on a sample of 7 mg, while recording weight loss during heating under air to 600 °C at a rate of 20 °C/min. The samples for FTIR analysis were embedded in KBr pellets and measured against a background record. The FTIR spectra were recorded with a resolution of 4 cm^−1^ and averaged over 32 scans. The Raman spectra were recorded with a near-infrared laser source of 785 nm and 40 mW power at the sample surface, an exposure time of 2 s for every 10 exposures, and resolution of 2 cm^−1^.

## 3. Results

### 3.1. Dispersion Properties and Morphology

The dispersion properties of synthesized aqueous wax nano-emulsions are evaluated in Table 1, including S.C., pH, viscosity, z-average particle size and zetapotential. Both variations in wax quantity and solid content of the emulsions influenced the physico–chemical properties. The pH of the emulsion could be seen as a good estimation for the yield of the imidization reaction, as the reaction involved first an increase in pH, owing to ammonolysis of the maleic anhydride (i.e., ring-opening reaction), followed by a decrease in pH upon completion of the imidization reaction (i.e., ring-closure of the anhydride five ring into imide structure). Therefore, lower pH values indicated better finalization of the imidization reaction. In parallel with the previous data for pure SMI [46], a slight acidic environment was created with 5 < pH < 6, owing to a residual fraction of non-imidized (i.e., ring-opened) maleic anhydride moieties. The negative charges around the ring-opened COO– functional groups both created a slight acidic environment in parallel with the stabilization of the emulsion through electrostatic repulsion effects. The low pH for pure SMI nanoparticles was not approached by the wax emulsions, as an indication of the interference of the imidization reaction in presence of wax. The pH for the carnauba emulsions at low S.C. was steadily lower compared to emulsions with higher S.C., as the reaction conditions and diffusion of reagent might be favored under low solid content. The pH of wax emulsion with slightly increased with an increasing amount of carnauba, due to possible hindrances of imidization. The latter was mainly observed at the highest carnauba wax concentrations (CW50-80/20). For paraffin wax emulsions, similar trends were observed for the influences of S.C. and wax content, but all the pH values were a little higher, compared to the carnauba wax emulsions. The higher pH values pointed towards less complete imidization and remaining intermediate reaction products. Therefore, it could be concluded that the compatibility of the carnauba wax type with the reaction conditions was better than for paraffin wax, as the latter caused more interference with the imidization process.

The quality of the wax emulsion was also determined by the viscosity and could be related to the pH. Compared to the pure SMI suspensions, the stable wax emulsions had lower viscosity, under the same conditions of S.C., owing to the lubricating properties of the wax. The viscosity obviously increased for emulsions with a higher solid content, but excess increase in viscosity occurred for emulsions with the highest pH. This indicated that the latter emulsions, with high wax content, were less homogeneous (e.g., formation of a two-phase system with separate wax and organic polymer phase caused an increase in viscosity), or contained intermediate reaction products (e.g., residual ammonolysed SMA increased charge interaction between the polymer chains with consequent higher viscosity). The evolution of viscosity during reaction indeed indicated progressive decrease in viscosity upon completion of the encapsulation and imidization reaction, while the first reaction step, with ammonolysis of SMA, introduced an increase in viscosity. The viscosity for paraffin wax emulsions was systematically higher, compared to the carnauba wax emulsions with the same wax concentration and S.C., indicating better compatibility of the carnauba wax with the organic phase during emulsification.

The z-average particle sizes confirmed the formation of nanoparticles for pure SMI dispersions (diameter around 100 nm) and wax emulsions, while the encapsulation of wax involved an increase in nanoparticle sizes. For both carnauba and paraffin waxes, comparable particle sizes were measured, with a trend for the formation of larger nanoparticles at higher wax concentrations. The reasons could be attributed to the following: *(i)* the encapsulation of a higher wax content, or *(ii)* a slight reduction in viscosity owing to the lubricating properties of the wax, which introduced a lower internal shear during synthesis in the aqueous environment. The viscosity effect on particle size was most expressed for carnauba wax emulsions with a higher solid content, where the particle size of 180 to 200 nm was lower than the particle size for emulsions with a lower solid content between 200 to 250 nm. The viscosity effect on particle size was also observed for paraffin wax emulsions with low solid content, but less so for paraffin wax emulsions with high solid content, as the latter became more instable. The stability of the wax emulsions was best monitored through the zetapotential values, ranging from very stable dispersions, for pure SMI nanoparticles, towards stabilized wax emulsions with wax concentrations up to 70 wt.%. Negative zetapotential values indicated the stability of the wax emulsions attributed to the presence of negative charges occurring near the residual ammonolysed SMA moieties. The zetapotential for wax emulsions became slightly lower compared to pure SMI nanoparticle dispersions, being an indication of the higher concentration of residual ammonolysed SMA with ring-opened anhydride moieties and negative charges around the carboxylic acid groups. This was further explained in relation with the calculation of the imidized content, indicating only partial imidization reactions for the most stable emulsions. Negative zetapotential provided long-term stability of the paraffin and carnauba wax nano-emulsions without observable phase-separation, even after long storage times of 2 years in the laboratory. Alternatively, the nano-emulsion formulations with rice bran wax, formed through homogenization, presented particle distribution below 100 nm (92.56 to 94.52 nm), with optimum charge distribution (−55.8 to −45.12 mV) and pH (6.79 to 6.92), but only remained stable for 8 weeks [47].

**Figure 2:**
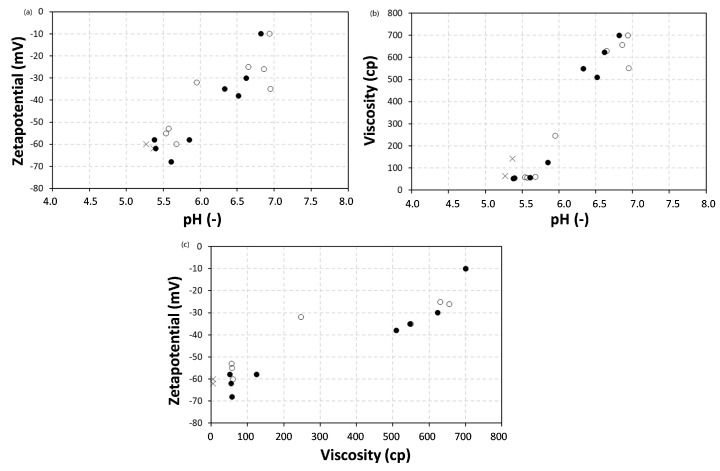
Relationships between physico–chemical properties of pure organic SMI particle dispersions (×), carnauba wax emulsions (●) and paraffin wax emulsions (o), including (**a**) Zetapotential versus pH, (**b**) Viscosity versus pH, (**c**) Zetapotential versus viscosity.

In summary, the relationships between physico–chemical properties of the wax emulsions are plotted in Figure 2, illustrating the trends discussed above. It could be concluded that stable emulsions were obtainable with a maximum concentration of 70 wt.-% bio-wax and S.C. = 50%. The latter conditions were selected as the best candidates for maximum wax content and best stability for both carnauba and paraffin wax. It is interesting that the physico–chemical properties for carnauba wax emulsions are closer to those of the pure SMI nanoparticle dispersions with lower pH, lower viscosity and lower zetapotential, compared to the paraffin wax emulsions.

**Figure 3:**
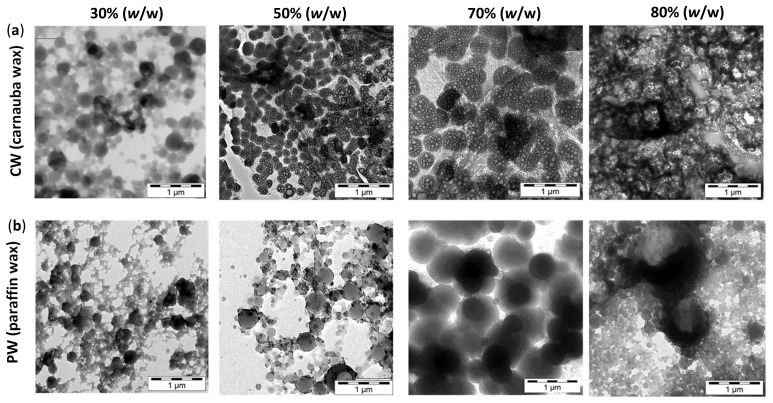
TEM observation of morphologies for wax nano-emulsions including carnauba wax and paraffin wax at different concentrations of 30, 50, 70, 80% (*w*/w) and fixed S.C. = 50%, (**a**) carnauba wax emulsions (top row), (**b**) paraffin wax (bottom row).

The morphology of wax emulsions was further evaluated by TEM (Figure 3), analyzing carnauba and wax emulsions with different wax concentrations and S.C. = 50%. The features for carnauba and paraffin wax emulsions were slightly different in parallel with the slight modifications in physico–chemical properties noticed before. The carnauba wax emulsions showed very good homogeneity in particle morphologies in parallel with formation of nanoscale particles with encapsulated wax. The carnauba wax was visible as single wax droplets encapsulated into the organic nanoparticles. The nanoparticle size increased in parallel with the increase in carnauba wax concentration and higher amounts of encapsulated wax. The emulsions with wax concentrations of 70% (*w*/w) formed optimized morphologies with the maximum content of emulsified wax. The higher wax concentrations of 80% (*w*/w) were not properly emulsified and a large volume of free wax could be visually observed as a continuous phase. The observed amount of encapsulated wax (70% *w*/w) was higher than previous methodologies for wax encapsulation in nano-emulsions obtained through mechanical sonication of molten wax in a surfactant solution, yielding maximum wax concentrations of 50% (*w*/w) [19]. The particle sizes for carnauba wax emulsions, according to TEM morphologies, were about 100–150 nm (30% *w*/w), 150–200 nm (50% *w*/w), to 200–250 nm (70% *w*/w), evidently increasing with wax concentration. It was also previously demonstrated that the size of capsules in the micron-scale range with encapsulated wax in a polymeric shell was controlled by the feeding ratio of polymer and wax [42]. The nanoparticle sizes were in agreement with previous DLS measurements, taking into account the effects of hydrodynamic particle diameter being somewhat larger in DLS measurements. The present particle sizes of carnauba wax emulsions were smaller compared to those obtained by a facile sonication route, developed to produce aqueous wax dispersion without any surfactants or stabilizers, resulting in particle sizes of 260 to 360 nm [48]. The paraffin wax emulsions demonstrated different particle morphologies with more heterogeneous particle sizes and less efficient encapsulation of the wax. The formation of particles with different morphologies was observed, including a fraction of particles with small sizes, together with a fraction of larger droplets. The smallest particles had sizes of 50–60 nm, while the size and quantity of larger droplets increased with the higher concentrations of paraffin wax. The large droplets had diameters of around 180–200 nm (30% *w*/w), 200–400 nm (50% *w*/w), to > 1 µm (70% *w*/w), representing the formation of non-encapsulated, or free, paraffin wax droplets. As observed for the droplets at highest wax concentrations of 70% (*w*/w), the wax seemed to protrude outside the particles with a dense core (darker area) and wax border around the particles (lighter area). This observation agreed with previous research, where a maximum encapsulation efficiency of 75% was reported for paraffin wax through in-situ polymerization of melamine–formaldehyde, with particle sizes of 260 to 450 nm [44]. In conclusion, the wax emulsions with carnauba wax formed more defined morphologies with smaller particles containing encapsulated wax within the organic polymer phase, while the paraffin wax provided phase separation, including large wax droplets.

### 3.2. Chemical Properties

**Figure 4:**
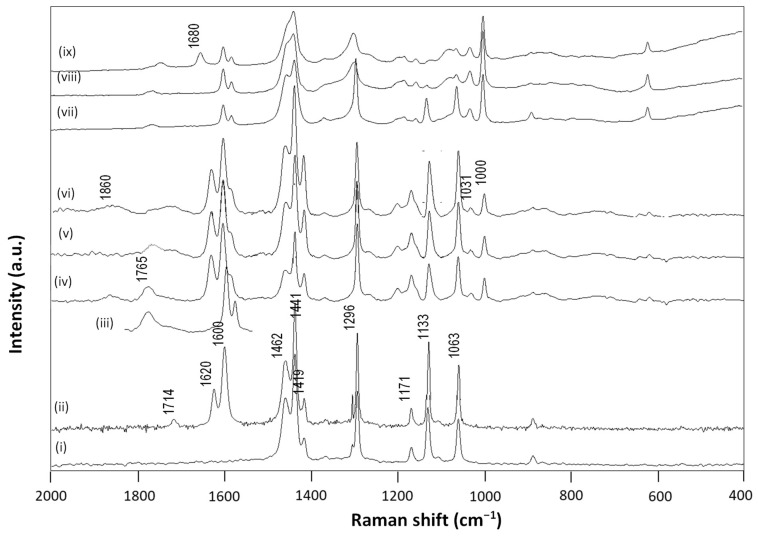
Raman spectra for the wax nanoparticles (dried from emulsion) and reference materials in the fingerprint region 2000 to 400 cm^−1^, including (i) pure paraffin wax (PW), (ii) pure carnauba wax (CW), (iii) pure SMI nanoparticles (iv) CW50-50/50, (v) CW50-70/30, (vi) CW50-80/20, (vii) PW50-50/50, (viii) PW50-70/30, (ix) PW50-80/20.

The fingerprint area of Raman spectra for some wax emulsions is shown in Figure 4, together with reference spectra for pure carnauba wax, paraffin wax, and SMI nanoparticles. Owing to the differences in intrinsic structures between carnauba wax and paraffin wax, including either more regular aliphatic hydrocarbon structures (n-paraffin), or a mixture of wax esters (carnauba wax), the Raman spectra for reference wax materials differed.

The Raman spectra of paraffin wax were characterized by distinct contributions of carbon–carbon stretching in the aliphatic polymer chains, including 1063 cm^−1^ (C-C skeletal stretch), 1133 cm^−1^ (C-C stretch), 1171 cm^−1^ (C-C stretch), 1296 cm^−1^ (CH_2_ deformation) and the triplet at 1419 cm^−1^ (CH_2_ deformation), 1441 cm^−1^ (CH_2_ deformation), 1462 cm^−1^ (CH_2_ bending and rocking) [49]. The paraffin wax was of very high purity with minor amounts of cyclo-paraffins contained in the wax (no C-C aromatic band at 1004 cm^−1^). The Raman peaks of paraffin wax at 1063 and 1171 cm^−1^ were weaker, compared to those of carnauba wax. Additionally, the Raman spectra of paraffin wax and carnauba wax exhibited very slight differences in peak positions in the range of 1400 to 1450 cm^−1^. The carnauba wax had additional Raman doublet bands at around 1600 to 1620 cm^−1^ related to the carbonyl stretching vibration (C=O) of ester or free acid components, which was an important distinct feature for plant wax. The latter region also overlapped with the presence of the C=C stretching mode at 1631 cm^−1^ (in cis configuration) and 1610 cm^−1^ (isolated). The presence of free fatty acids was confirmed by the band at 1714 cm^−1^ (C=O) [50].

A reference spectrum for the imidized styrene–maleic anhydride showed characteristic Raman bands at 1765 cm^−1^ (imide I, C=O stretching) and a doublet at 1602, 1583 cm^−1^ (styrene, aromatic C=C) [46]. The original SMA copolymers were characterized by the presence of maleic anhydride (1860 cm^−1^, C=O anhydride) that gradually disappeared after ammonolysis and imidization. Only this region is illustrated, as it had less overlap with the other functional groups of the wax and allowed for further quantification of the imidization reaction, as illustrated before for the pure SMI nanoparticles [46]. The Raman spectra illustrated very well the efficient formulation of wax nano-emulsions in combination with imidized styrene (maleic anhydride), with a more favorable formation of imide structures in the presence of carnauba wax. The Raman spectra for (iv) CW50-50/50 and (v) CW50-70/30 had clear bands related to the formation of the imide moieties, in parallel with good retention of the carnauba bands up to wax concentrations of 70% (*w*/w). The Raman spectrum for (vi) CW50-80/20 with high wax concentrations had lower intensities of bands related to imide structures and there were significant remnants of the maleic anhydride (1860 cm^−1^) and ring-opened carboxylic acid moieties (1860 to 1750 cm^−1^). The wax emulsions also indicated the presence of styrene moieties originating from the SMA copolymer at Raman bands of 1031 and 1000 cm^−1^. The observed reversion in relative intensity for the wax Raman bands at 1063 and 1133 cm^−1^ could be attributed to the overlap with styrene Raman bands. This was seen for both the carnauba and the paraffin wax emulsions, and could be related to non-reactive hydrocarbon CH_2_ moieties in the wax. Alternatively, the slight variations in relative intensities could relate to conformational constraints of the aliphatic chains after reaction and (partial) binding to the imidized organic phase. The latter, however, was less likely for the n-paraffin wax containing no reactive sites in the aliphatic chain. The reduced efficiency for encapsulation and stabilization of paraffin wax emulsions was clearly seen in the Raman spectra (vii) PW50-50/50, (viii) PW50-70/30, with strong alterations in the characteristic wax Raman bands at 1400 to 1460 cm^−1^ and the disappearance of the Raman bands at 1063 and 1133 cm^−1^ after emulsification. In parallel, the imide content was lower and a significant portion of free carboxylic acids (1680 cm^−1^) remained present in the Raman spectra (ix) PW50-80/20 for paraffin wax concentrations of 80% (*w*/w).

**Figure 5:**
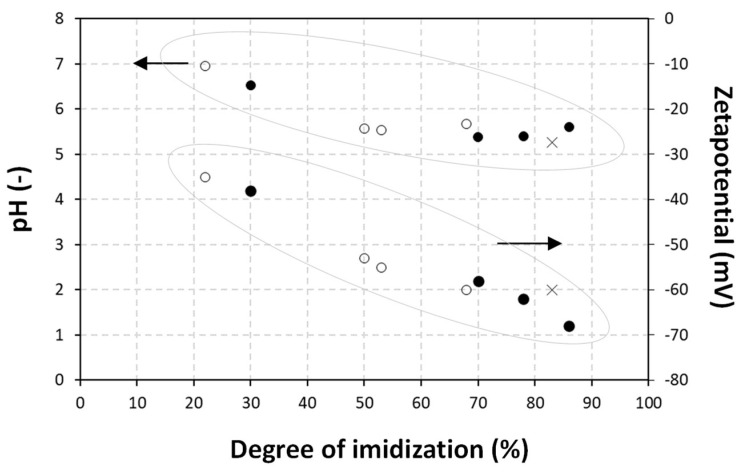
Relationship between chemical properties (i.e., degree of imidization) of the wax nanoparticles and physico–chemical properties of the wax emulsions, including pure SMI nanoparticles as an organic phase (×), carnauba wax (CW) emulsions (●) and paraffin wax (PW) emulsions (o).

Based on Raman spectroscopy, analytical quantification of the degree of imidization and encapsulated wax is presented in Table 2, offering an estimation for the efficiency for wax emulsification. The imidization reaction could be quantified in terms of imide content, i.e., the amount of formed imide groups relative to the styrene moieties. The latter could be calculated from the relative ratio of Raman band intensities of imide (1765 cm^−1^) to styrene (1602 cm^−1^) [40]. The maximum imide content for the selected SMA copolymer, with 26 mol-% maleic anhydride groups, equaled 35%, as calculated from the fact that all anhydride groups were converted into the imide structure according to the ratio 26/(26 − 100) = 0.35. The degree of imidization was then expressed as the imide content after reaction relative to the maximum theoretical imide content. However, the maximum degree of imidization was a theoretical event where the stability of the SMI nanoparticle dispersion would collapse in the absence of a residual amount of ammonolysed SMA. The latter acted as a surfactant with negative charge stabilization of the dispersions, as previously confirmed by the negative zetapotential values. Alternatively, the amount of encapsulated wax could be calculated from the relative ratio of the Raman band intensities of wax (1063, 1133 cm^−1^) to styrene (1602 cm^−1^). As an illustration for cross-validation of the analytical methodologies used in this research, the relationships between degree of imidization and physico–chemical emulsion properties are illustrated in Figure 5, confirming the effects of a fraction of non-imidized maleic anhydride moieties on the emulsion stability, as discussed before.

**Figure 6:**
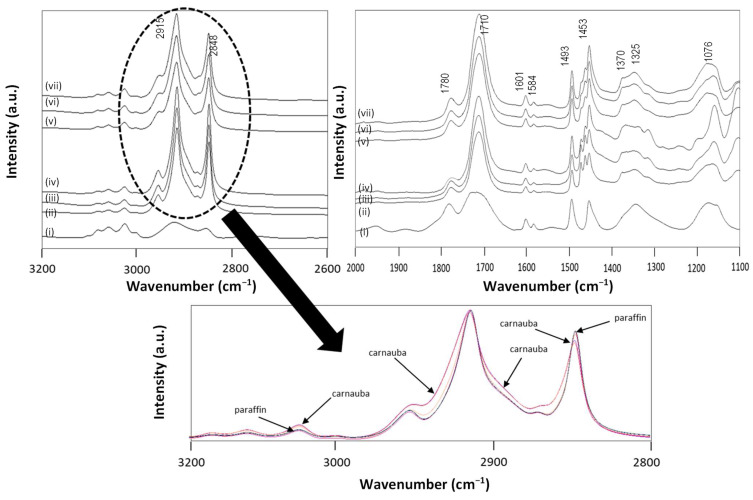
FTIR spectra for the wax nanoparticles (dried from emulsion) and reference materials, including (i) pure SMI nanoparticles (ii) CW50-50/50, (iii) CW50-70/30, (iv) CW50-80/20, (v) PW50-50/50, (vi) PW50-70/30, (vii) PW50-80/20.

The FTIR spectra for some wax emulsions and a reference of pure SMI nanoparticles, are illustrated in Figure 6. The FTIR bands for imidized styrene-maleic anhydride were localized at 1780 cm^−1^ (symmetric C-O, imide I); 1710 cm^−1^ (asymmetric C-O or N–C–O stretch, imide I); 1601, 1584 cm^−1^ (styrene, C–C stretch); 1493, 1453 cm^−1^ (styrene, aromatic C–C stretch); 1370, 1325 cm^−1^ (styrene, C–H aromatic vibrations); 1076 cm^−1^ (cyclic C–O–C and carbonyl, anhydride) [46]. The FTIR reference spectra for paraffin wax and carnauba wax had characteristic bands for both wax types at 2915 and 2848 cm^−1^, corresponding to the asymmetric and symmetric stretching vibration of C–H from methyl and methylene groups, respectively [51]. A very slight variation in the conformation around those bands was seen after incorporating carnauba wax (detailed overlay in Figure 6), indicating eventual constraints on the long aliphatic chains after reaction with the organic phase. In parallel, higher interactions through hydrogen bonding between the organic phase and the carnauba wax were observed, owing to the presence of ester functionalities in the carnauba wax. The paraffin wax had sharp bands associated with the saturated hydrocarbon linear polymer chains, including CH_2_ bend or scissoring deformation (1466 cm^−1^), CH_3_ symmetric deformation (1380 cm^−1^) and CH_2_ rocking deformation of the long-chain alkenes (720 cm^−1^) [52]. The carnauba wax had an additional band at 1730 cm^−1^, corresponding to the presence of ester and diesters [53]. The carnauba wax indeed consisted of multiple chain length fatty acid esters, fatty alcohols, acids and hydrocarbons, mainly including C_26_ acid and C_32_ alcohol. The presence of wax in the emulsified samples was confirmed by the appearance of the corresponding FTIR bands in the spectra, but full quantitative analysis could not be conducted, due to strong overlap in the different functionalities. The gradual increase in wax concentrations for the different samples could be confirmed with a rise in the corresponding FTIR bands (e.g., 1466 cm^−1^). The additional information from FTIR analysis, compared to Raman analysis, was situated in the confirmation of the formation of amic acid moieties for the wax concentrations at 80% (*w*/w), seen in the broad absorption region with slightly increased intensities at 1580 to 1560 cm^−1^ (amide II, N–H bending), and 1412 cm^−1^ (amide stretching mode in amic acid), corresponding to acid moieties (COOH, CONH_2_) from ring-opened anhydride.

### 3.3. Thermal Properties

**Figure 7:**
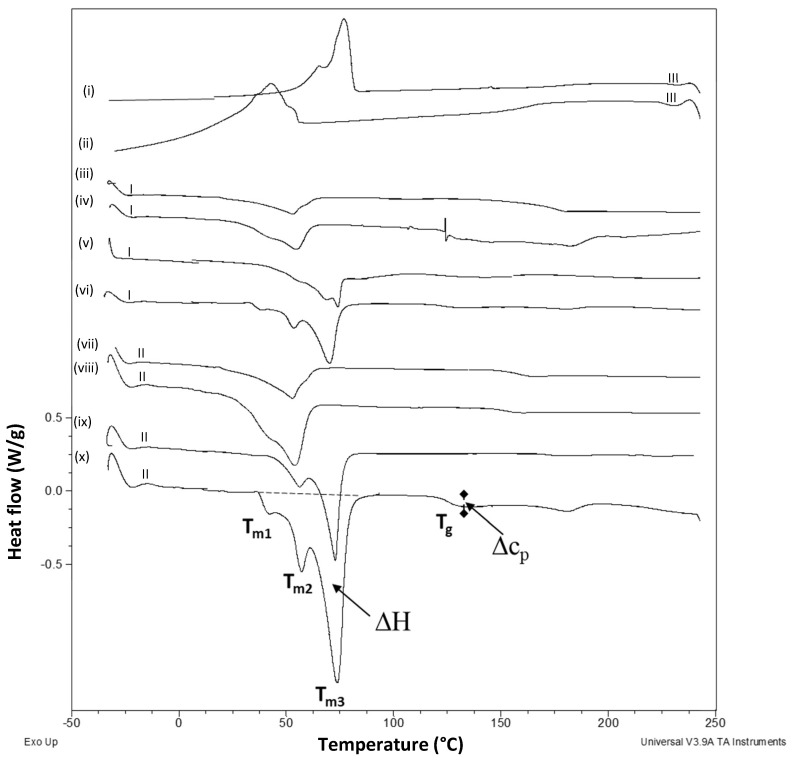
Illustration of DSC curves for different wax nanoparticles (dried from emulsion) during first heating cycle (I) and second heating cycle (II) with intermediate cooling step (III), including (i) cycle III for CW50-50/50, (ii) cycle III for PW50-50/50, (iii) cycle I for PW50-50/50, (iv) cycle I for PW50-80/20, (v) cycle I for CW50-50/50, (vi) cycle I for CW50-80/20, (vii) cycle II for PW50-50/50, (viii) cycle II for PW50-80/20, (ix) cycle II for CW50-50/50, (x) cycle II for CW50-80/20.

The DSC curves for some wax particle compositions are shown in Figure 7, including first heating and second heating curves with intermediate cooling and crystallization steps. Further quantitative data from DSC analysis are given in Table 3. The heating curves were characterized by the melting temperatures *T_m_* of the paraffin or carnauba wax and glass transition temperatures *T_g_* of the imidized SMA. The cooling curves indicated the crystallization behavior of the respective waxes, as the imidized SMA remained in the amorphous phase. Both transitions were observed in the thermographs with some variations in intensity of the wax melting transitions between the first and second heating samples, indicating a larger melting enthalpy Δ*H* for the wax in the second heating cycle and a possible shift in *T_g_* of the polymer phase. These could be related to the partial release of wax from the polymer phase during the first heating step and reduced constraints in the polymer phase, lowering the *T_g_*, or decreasing the intensity of the transition, owing to restricted phase mobility. In parallel with thermal characterization of micron-scale wax particles in previous studies [42], the melting enthalpy decreased for a higher amount of organic phase functioning as a shell structure, while the melt and crystallization temperatures had almost no change. Therefore, an almost linear correlation could be found when calculating the core content from the enthalpy. The crystallization phenomena in the cooling cycle were within expectations for both wax types.

The pure paraffin wax had a melting point of *T_m_* = 55.8 °C, corresponding to the major solid–liquid transition and a minor shoulder peak at *T_m_* = 45.5 °C, related to a minor solid–solid phase change (reorganization of orthorhombic into hexagonal lattice). The same transitions were observed for the encapsulated wax materials, but the intensity of the solid–solid transition was less pronounced during the first heating step and at concentrations of up to 70% (*w*/w), illustrating some constraints between the wax and polymer phase. The first solid–solid transition became more pronounced at higher wax concentrations of 80% (*w*/w), owing to the presence of excess free wax that was not encapsulated. The suppression of the solid–solid transition in paraffin wax, in the presence of non-imidized styrene maleic anhydride, was demonstrated before in [42]; as the latter was added as a mechanical strengthening agent that reduced the softness of the wax. Moreover, anhydride copolymers and derivatives are known as wax crystal modifiers, where the polymeric derivative with nitrogen-containing short branches along the polymer backbone (e.g., comparable to imidized groups) perform better than the introduction of short-chain esters [54]. The formation of complex intermediate structures between the free wax and the non-imidized SMA copolymer induced strong irregularities in the heating curves (mainly the first heating step) and a reduction in the glass transition temperature between the first and second heating cycles, from *T_g_* = 160 °C towards *T_g_* = 150 °C. The higher concentrations of paraffin wax introduced a further reduction in *T_g_* from 150 °C to 145 °C. As a reference, the pure organic phase of SMI nanoparticles had a glass transition temperature of *T_g_* = 180 °C [46], and a reduction in *T_g_*, which agreed with the previously calculated low degree of imidization. The melting temperatures of the presented nanocapsules were higher than other paraffin wax nanocapsules with melamine-formaldehyde shells, where a melting temperature of *T_m_* = 49 °C was similar to that of the pure paraffin wax [44].

**Figure 8:**
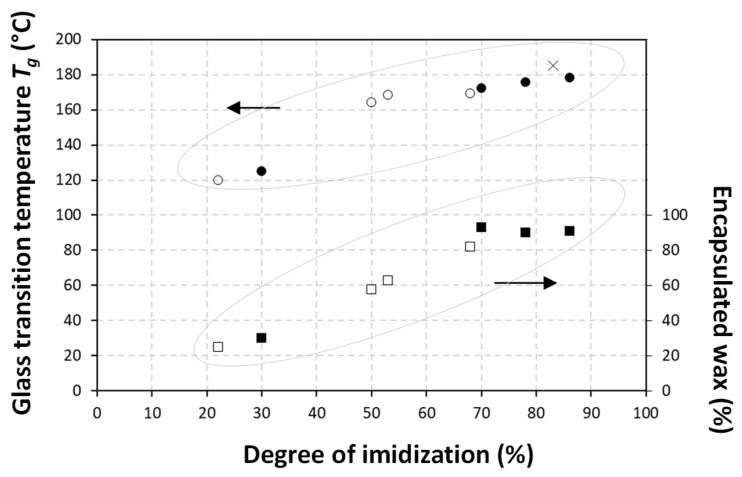
Relationship between thermal properties and chemical properties (i.e., degree of imidization and encapsulated wax) of the wax nanoparticles, including pure SMI nanoparticles as an organic phase (×), carnauba wax (CW) emulsions (●, ■) and paraffin wax (PW) emulsions (o, □).

The pure carnauba wax had a more complex melting trajectory, with higher melting temperature, as it is one of the highest melting natural waxes and can, therefore, be well distinguished from other waxes. The differences in thermal transitions could be related to its particular composition, which mainly contains fatty acid esters (70 to 82%), free alcohols (8 to 14%) and free acids (1 to 4%) and hydrocarbons (1 to 6%), with each having a specific melting behavior [55]. It was demonstrated before for pure carnauba wax that the first melting endotherms at *T_m_* = 61 and 78 °C were attributed to the heat absorbed by free acids and hydrocarbons, while the dominant melting endotherm at *T_m_* = 83 °C corresponded to heat absorption by fatty acid esters. In the present case of encapsulated carnauba wax, the main melting endotherm dropped to *T_m_* = 74 to 75 °C, illustrating efficient interactions between the carnauba wax and organic phase. The suppression of melting temperatures for wax emulsions in respect to the bulk solid wax could indeed be expected, as it was similarly observed in other studies that the melting phenomena of encapsulated wax diminished after the addition of carvacrol, or polymeric substances, as nanocarrier materials [56]. Comparing the first and second heating cycles, the fractionated melting steps of different wax components became clearer in the second run, as part of the wax was released and re-crystallized after the first heating and cooling cycles. The weak melting transition during the first heating cycle was, thus, an illustration of the encapsulation of wax and suppressed melting. In particular, the first melting peaks related to free acids were less resolved and had lower intensity in the first heating cycle, as the latter could be expected to be most active in reaction with the organic phase. The favorable interactions between the carnauba wax and the organic phase were also reflected in a reduction in the heat capacity change for carnauba concentrations up to 70% (*w*/w). The *T_g_* related to the organic phase for nanoparticles with carnauba wax were less pronounced than for nanoparticles with paraffin wax, confirming more interactions and constraints in the presence of carnauba wax. The transitions for the higher wax concentrations were clearly disturbed through the presence of free wax and severe hindrance of the imidization, with the effect of the remaining ammonolysed maleic anhydride moieties leading to a reduction in glass transition temperature *T_g_* = 120 °C. The relationships between the calculated degree of imidization and thermal properties of the wax nanoparticles are presented in Figure 8, confirming a good cross-validation of methodologies and verification of the findings obtained in this research.

**Figure 9:**
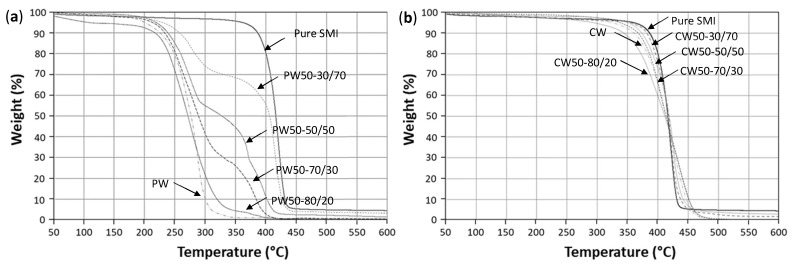
Thermal stability evaluated by TGA measurements for (**a**) paraffin wax (PW) nanoparticles, (**b**) carnauba wax (CW) nanoparticles.

The TGA curves with weight loss (%) for paraffin wax nanoparticles (Figure 9a) and carnauba wax nanoparticles (Figure 9b) are shown in the overlap of the original waxes and pure SMI nanoparticles. As a reference for the SMI nanoparticles, a residual weight fraction remains presented in agreement with our previous reports, owing to the formation of an oxidized network that fully degraded above 600 °C. The thermal stability of pure paraffin wax was obviously lower than carnauba wax [57,58], with main decomposition temperatures of *T_d,paraffin_* = 270 °C and *T_d,carnauba_* = 420 °C. For paraffin wax nanoparticles, two separated degradation steps were observed due to the following: *(i)* the wax phase (fixed position between 220 and 320 °C), and *(ii)* the organic phase (variable position depending on the degree of imidization), with weight loss fractions in relation to the paraffin wax concentrations (i.e., 28.5 % for 30% (*w*/w) paraffin, 48.2% for 50% (*w*/w) paraffin, 70.5% for 70% (*w*/w) paraffin, 88.1% for 80% (*w*/w) paraffin). It could be noticed that the weight loss during the first degradation step was roughly within the range of the present wax concentration, except for the highest wax concentrations where the structure became severely distorted, as illustrated above. Similarly, the composition of binary paraffin wax mixtures or wax polymer blends could be determined, depending on the partial decomposition of both phases [59]. Although the degradation temperatures for carnauba wax and the pure SMI nanoparticles became close to each other, the broader degradation interval for the carnauba wax was expected, depending on its more complex composition, with species of different molecular weight. In literature, the binary mixtures of a carnauba wax with polymers resulted in a two-step degradation pattern with weight losses corresponding to the carnauba wax concentrations, and the highest degradation temperature at 400 to 420 °C corresponding to the virgin carnauba wax degradation [60]. However, the same quantitative analysis could not be successfully made for the present carnauba wax nanoparticles, as more chemical interactions between the carnauba wax and imidized organic phase were demonstrated in the analysis presented earlier. However, for the nanoparticles with both carnauba and paraffin wax concentrations of 80% (*w*/w), the thermal stability strongly decreased and significant weight losses were already observed at temperatures below the main degradation step, likely in parallel with the degradation of intermediates or non-imidized moieties in parallel with the previous calculations indicating a low degree of imidization (see Table 2).

**Figure 10:**
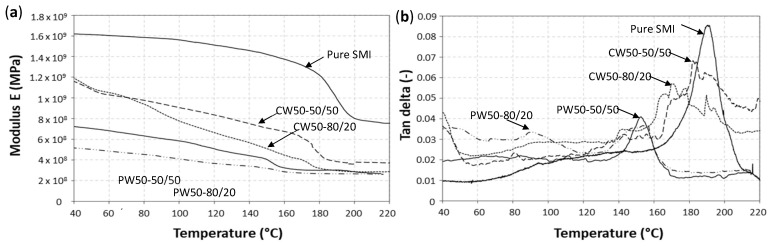
Thermal stability evaluated by DMA measurements for paraffin wax (PW) and carnauba wax (CW) nanoparticles, (**a**) modulus E, (**b**) dampening factor tan δ.

The DMA curves for paraffin wax and carnauba wax nanoparticles were characterized by measurement of the modulus (Figure 10a) and dampening factor tan δ (Figure 10b), showing values only interpretable as a relative comparison, rather than exact numerical data, owing to the applied testing method. As the powdery particles were sheared within a steel envelope, the latter predetermined the mechanical properties (e.g., exact numerical values of modulus and tan δ), while good relative sensitivity was observed reflecting the properties of the embedded soft polymer layer with induced shear under bending. The glass transitions were clearly observed as a drop in modulus and maximum in tan δ, often being more sensitive under dynamic mechanical conditions compared to static DSC, and certainly so for systems with complex phase interaction. The relative stiffness was indeed highest for the pure SMI nanoparticles with *T_g_* = 185 °C being in agreement with the DSC values above. The carnauba wax nanoparticles had *T_g_* = 178 °C (CW50-50/50) or *T_g_* = 175 °C (CW50-80/20) corresponding to the behavior of the organic phase, in line with previous DSC analysis. The presence of carnauba wax caused progressive softening at temperatures below *T_g_*, owing to the lubricating properties of the wax. The mechanical properties in the presence of carnauba wax were higher, compared to paraffin wax, in parallel with previous demonstrations of better compatibility and interactions between the organic phase and wax phase. The paraffin wax introduced mechanical softening and a lower *T_g_* = 153 °C (PW50-50/50), while the glass transition became smoother as the paraffin wax concentration was further augmented (PW50-80/20). The progressive melting of the wax below *T_g_* of the organic phase could be identified more clearly in the multiple tan δ transitions, indicating a trend for a higher dampening factor below *T_g_* as the wax concentration increased. The variations in tan δ around *T_g_* for carnauba wax nanoparticles was more complex than for paraffin wax nanoparticles, as a final confirmation for the multiple interactions between carnauba wax and the organic phase. 

## 4. Conclusions

Biowax nano-emulsions in an aqueous environment can be prepared through the encapsulation of carnauba wax, by means of an in-situ imidization reaction in the presence of an organic phase of SMA copolymers. The organic phase provides both physico–chemical stabilization of the emulsion and high thermal stability. The properties of the biowax emulsion can be particularly controlled by the reaction conditions of the imidization process towards the desired degree of imidization. Owing to the intrinsic structure of the biowax, relative to traditional paraffin wax, the carnauba wax presented higher reactivity, efficiency for encapsulation, and compatibility with the imidization process, offering a higher amount of encapsulated wax and a higher degree of imidization. A maximum concentration of 70% (*w*/w) carnauba wax could be incorporated forming porous nanoparticles of 200 nm with encapsulated wax droplets.

The presented method illustrates a pathway to provide stable aqueous biowax emulsions that are of high significance for several industries, in particular for developing protective coatings. The future upscaling of the synthesis procedures from current laboratory-scale conditions (1 L reactor) to industrial conditions is foreseen to be successful, in parallel with previous experiences of industrial synthesis of comparable nanoparticle emulsions at large scale, considering good control of critical parameters, such as temperature distribution and shear conditions, within the larger reaction medium. The fully waterborne synthesis route of biowax nano-emulsions and compatibility with waterborne coating formulations may offer future perspectives for application of the biowax as additives in coatings, e.g., targeting wood and paper coating industry. The long-term stability of the emulsions, owing to the anionic stabilization without the addition of external stabilizers, is expected to ensure good compatibility with existing coating formulations and to provide limited migration throughout the coating. While a generalized concept was presented herein, the latter coating properties need to be further investigated in relation with the type of encapsulated biowax, application conditions and post-treatments.

## Figures and Tables

**Table 1:** Physico–chemical characterization of SMI nanoparticle dispersions (no wax) and wax emulsions with carnauba wax (CW) and paraffin wax (PW). Presentation of the testing matrix with emulsions containing different wax concentrations and solid contents.

Sample * Ratio wax/SMA	Solid Content (S.C.) (% *w*/w)	pH	Viscosity (cp)	Z-Average Particle Size (nm)	Zeta Potential (mV)
No wax					
SMI	50	5.27	64	98	−60
SMI	60	5.37	143	90	−62
Carnauba wax					
CW50-30/70	50	5.61	57	206	−68
CW50-50/50	50	5.40	55	218	−62
CW50-70/30	50	5.38	50	250	−58
CW50-80/20	50	6.52	510	N/A	−38
CW60-30/70	60	5.85	125	180	−58
CW60-50/50	60	6.33	548	189	−35
CW60-70/30	60	6.62	623	196	−30
CW60-80/20	60	6.82	>700	N/A	>−10
Paraffin wax					
PW50-30/70	50	5.68	60	200	−60
PW50-50/50	50	5.54	58	230	−55
PW50-70/30	50	5.57	54	258	−53
PW50-80/20	50	6.95	550	N/A	−35
PW60-30/70	60	5.95	247	206	−32
PW60-50/50	60	6.65	630	220	−25
PW60-70/30	60	6.86	656	360	−26
PW60-80/20	60	6.94	>700	N/A	>−10

* Sample name includes S.C. and ratio wax/SMA in % (*w*/w).

**Table 2:** Quantification of encapsulated wax and degree of imidization for different wax nano-emulsions containing carnauba wax or paraffin wax in various concentrations, based on Raman spectroscopy.

	Sample	Encapsulated Wax (%)	Degree of Imidization (%)
No wax	SMI	-	83
Carnauaba wax	CW50-30/70	91	86
	CW50-50/50	90	78
	CW50-70/30	93	70
	CW50-80/20	30	30
Paraffin wax	PW50-30/70	82	68
	PW50-50/50	63	53
	PW50-70/30	58	50
	PW50-80/20	25	22

**Table 3:** Quantification of thermal properties according to DSC analysis.

Sample	First DSC Heating Cycle						Second DSC Heating Cycle					
	*T_m1_* (°C)	*T_m2_* (°C)	*T_m3_* (°C)	Δ*H* J/g	*T_g_* (°C)	Δ*c_p_* J/(g°C)	*T_m_*_1_ (°C)	*T_m_*_2_ (°C)	*T_m_*_3_ (°C)	Δ*H* J/g	*T_g_* (°C)	Δ*c_p_* J/(g°C)
No wax												
SMI	-	-	-	-	182.5	0.279	-	-	-	-	185.3	0.701
Pure Carnauaba wax (CW)	61.4	78.3	83.4	188.7	-	-	61.5	78.5	84.1	185.2	-	-
CW50-30/70	-	52.8	74.8	48.2	174.3	0.11	-	53.2	74.1	35.2	178.3	0.15
CW50-50/50	-	52.4	74.0	50.3	175.3	0.09	-	54.8	74.2	47.2	175.9	0.07
CW50-70/30	40.1	49.8	72.8	54.2	172.2	0.10	43.2	55.3	72.5	82.1	172.3	0.05
CW50-80/20	39.4	48.1	7.1	80.4	unclear	unclear	39.6	53.3	73.3	118.1	125	0.368
Pure Paraffin wax (PW)	-	-	55.8	49.8	-	-	-	-	55.4	49.6	-	-
PW50-30/70	-	-	53.8	12.3	171.8	0.123	-	-	53.5	18.4	169.3	0.25
PW50-50/50	-	-	53.1	12.8	171.2	0.117	-	-	52.9	23.7	168.7	0.19
PW50-70/30	-	41.1	53.8	13.1	166.3	0.111	-	41.3	53.5	35.2	164.2	0.18
PW50-80/20	-	40.8	53.4	28.4	unclear	unclear	-	40.5	53.2	45.7	120.2	0.15

## Data Availability

Not applicable.
